# Supplementary material for: Peptide Mediated Adhesion to Beta-Lactam Ring of Equine Mesenchymal Stem Cells: A Pilot Study
Source: Animals (Basel). 2022 Mar 15;12(6):734. doi: 10.3390/ani12060734 (PMC8944785; doi:10.3390/ani12060734)

Supplementary figure S1: panel for Figure 1 A - B

Coating

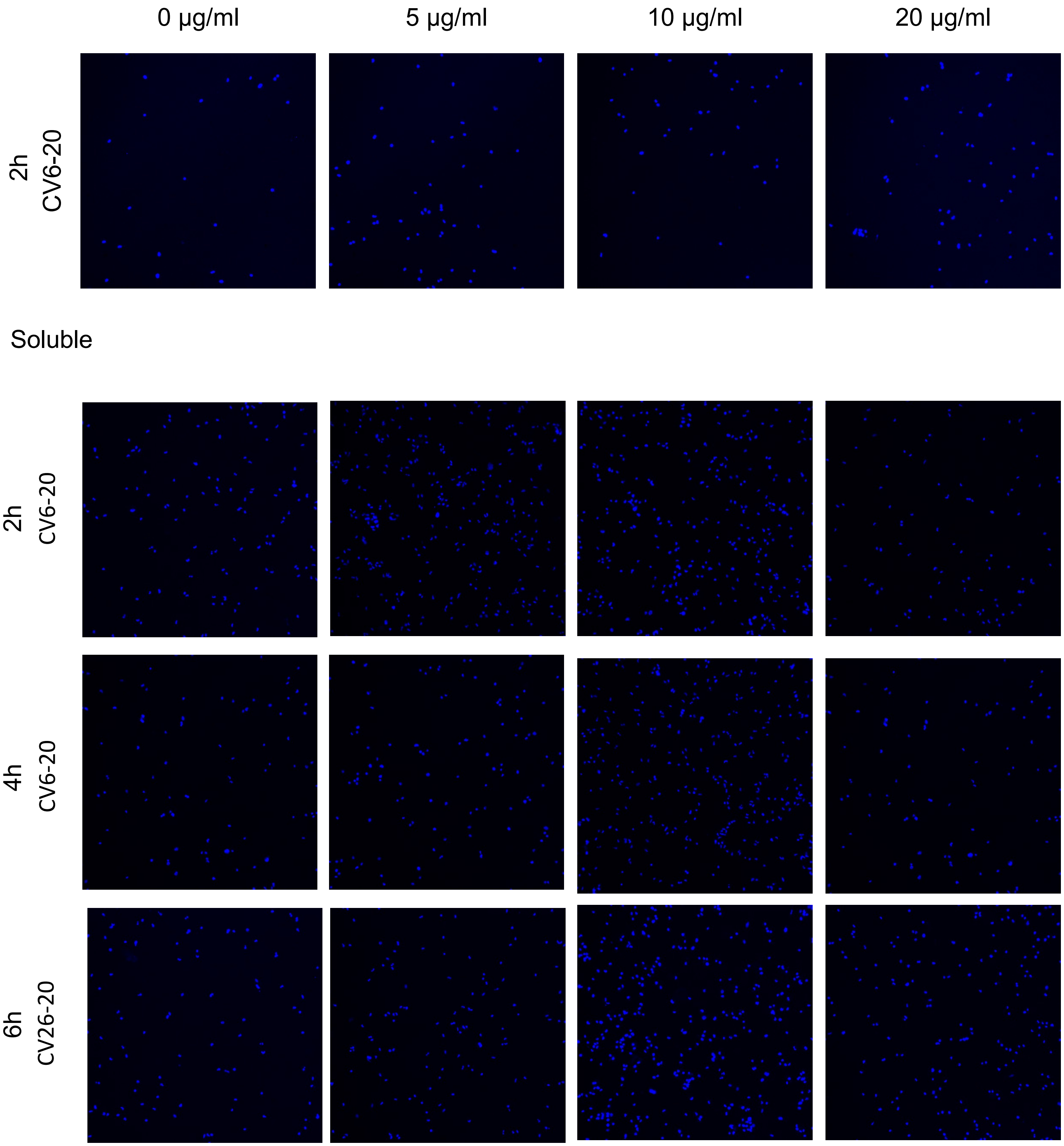

Supplementary Figure S2: panel for Figure 1 C - F

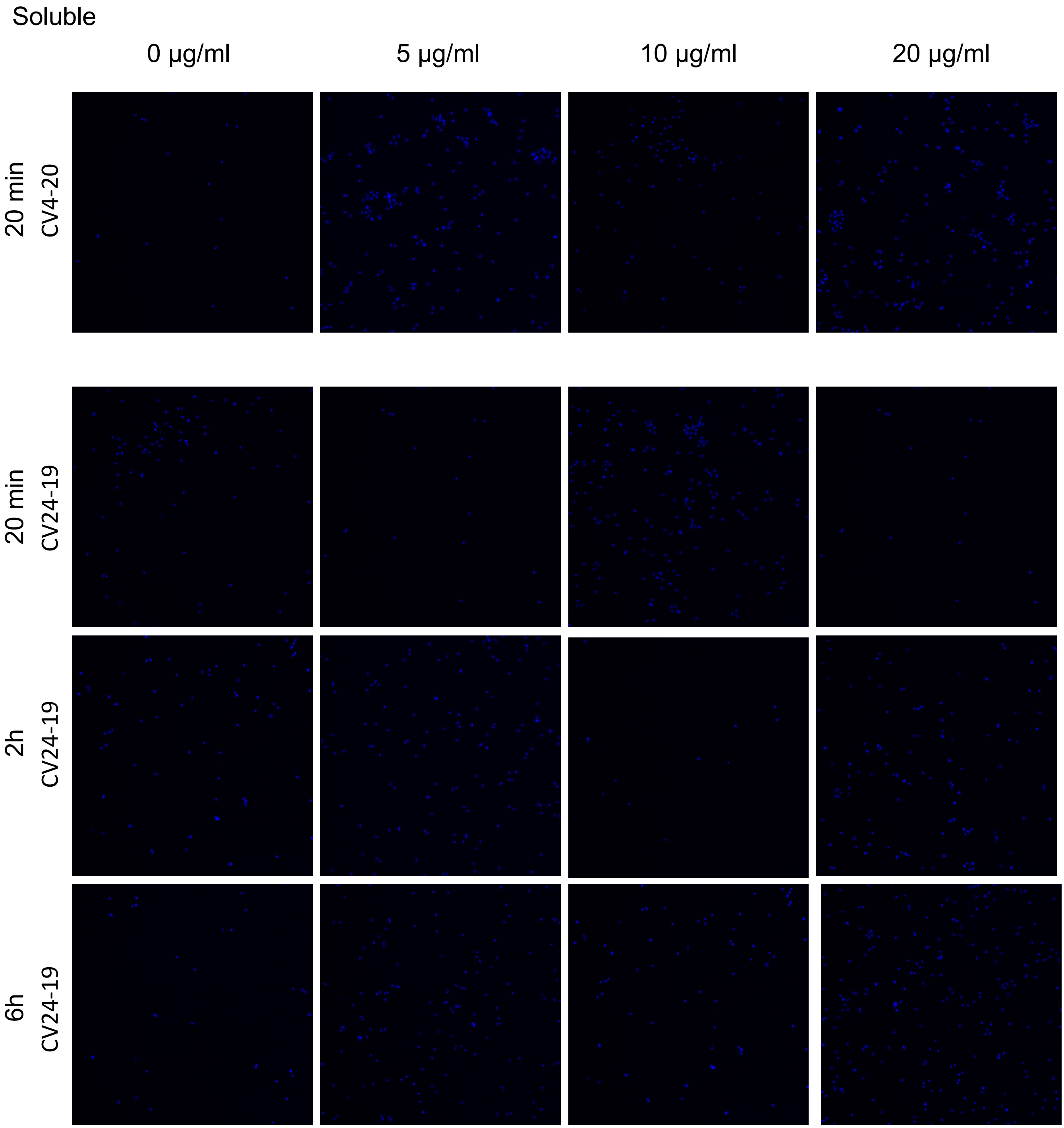

Supplementary Figure S3: panel for Figure 2 B

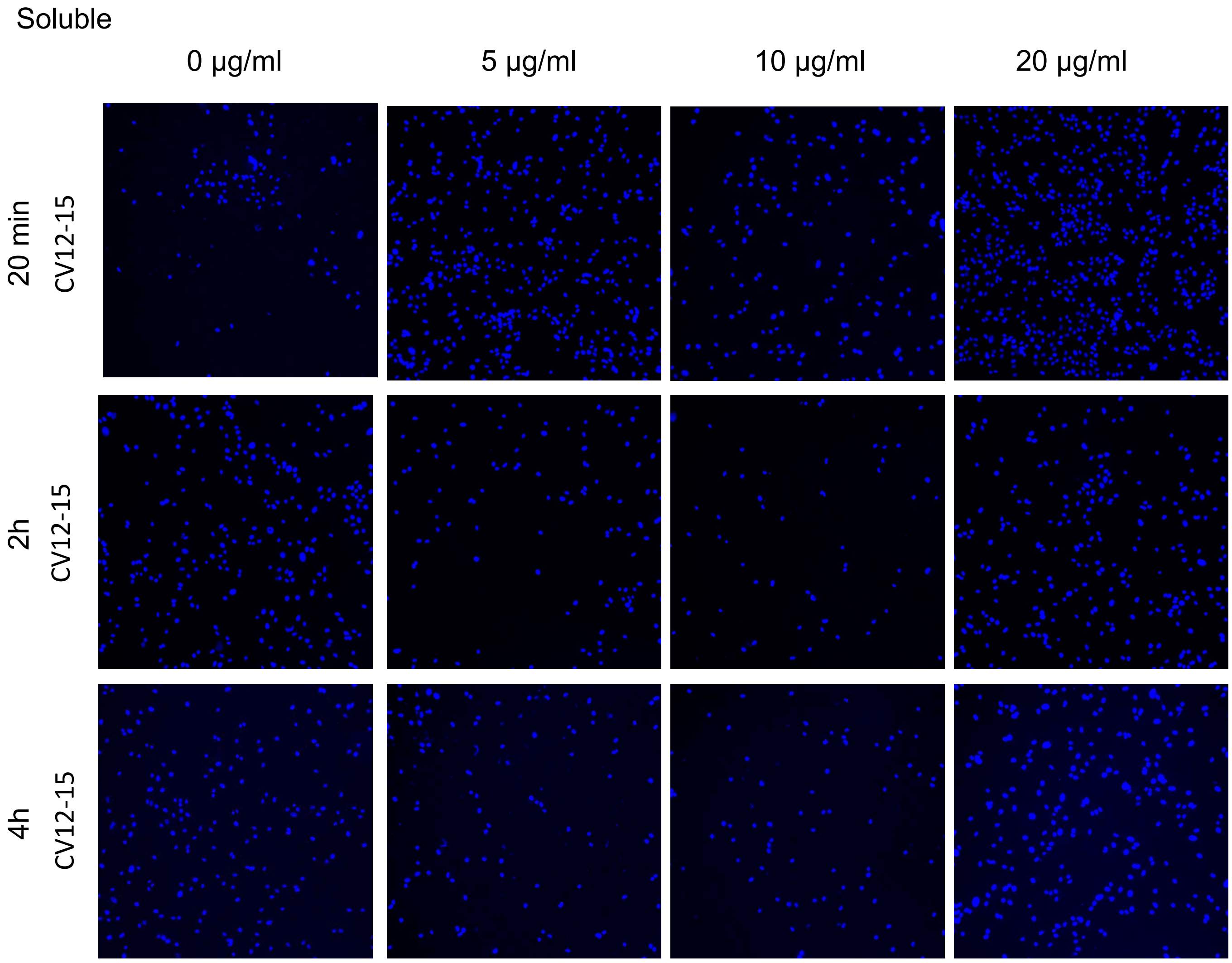

Supplementary Figure S4: panel for Figure 2 D

Soluble

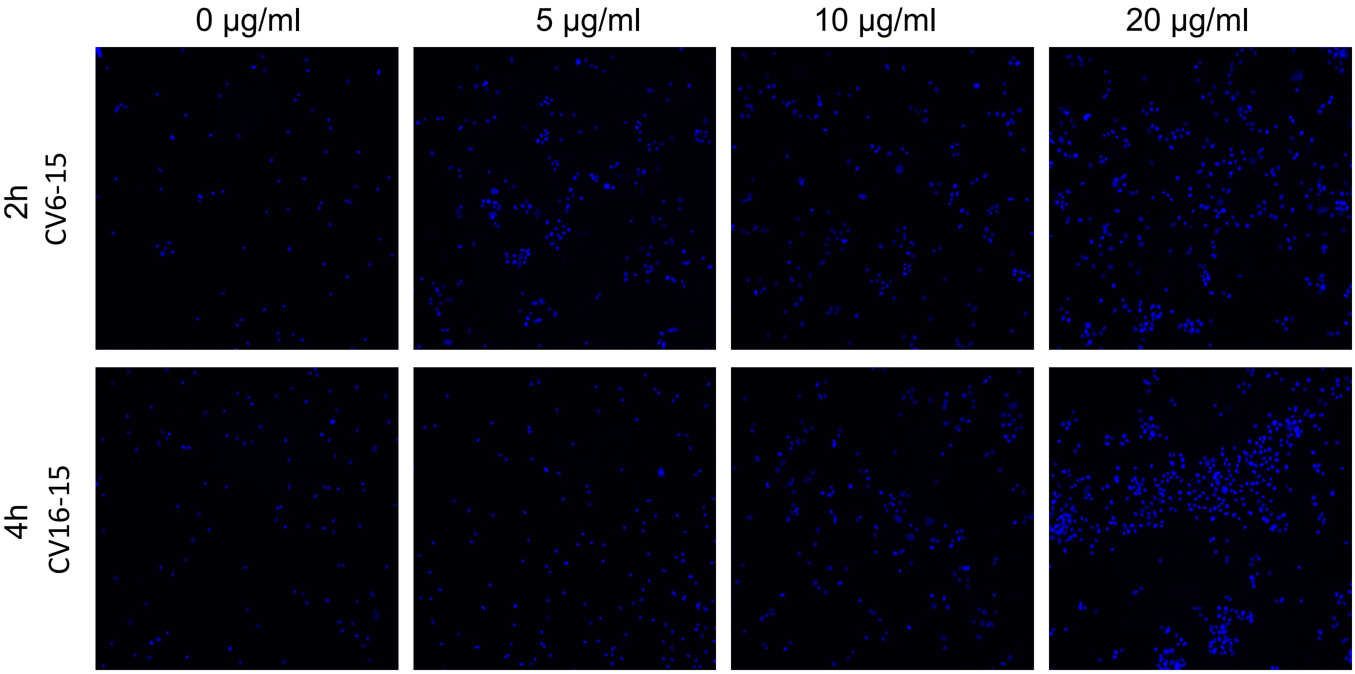

Supplementary: panel for Figure 2 F

Soluble

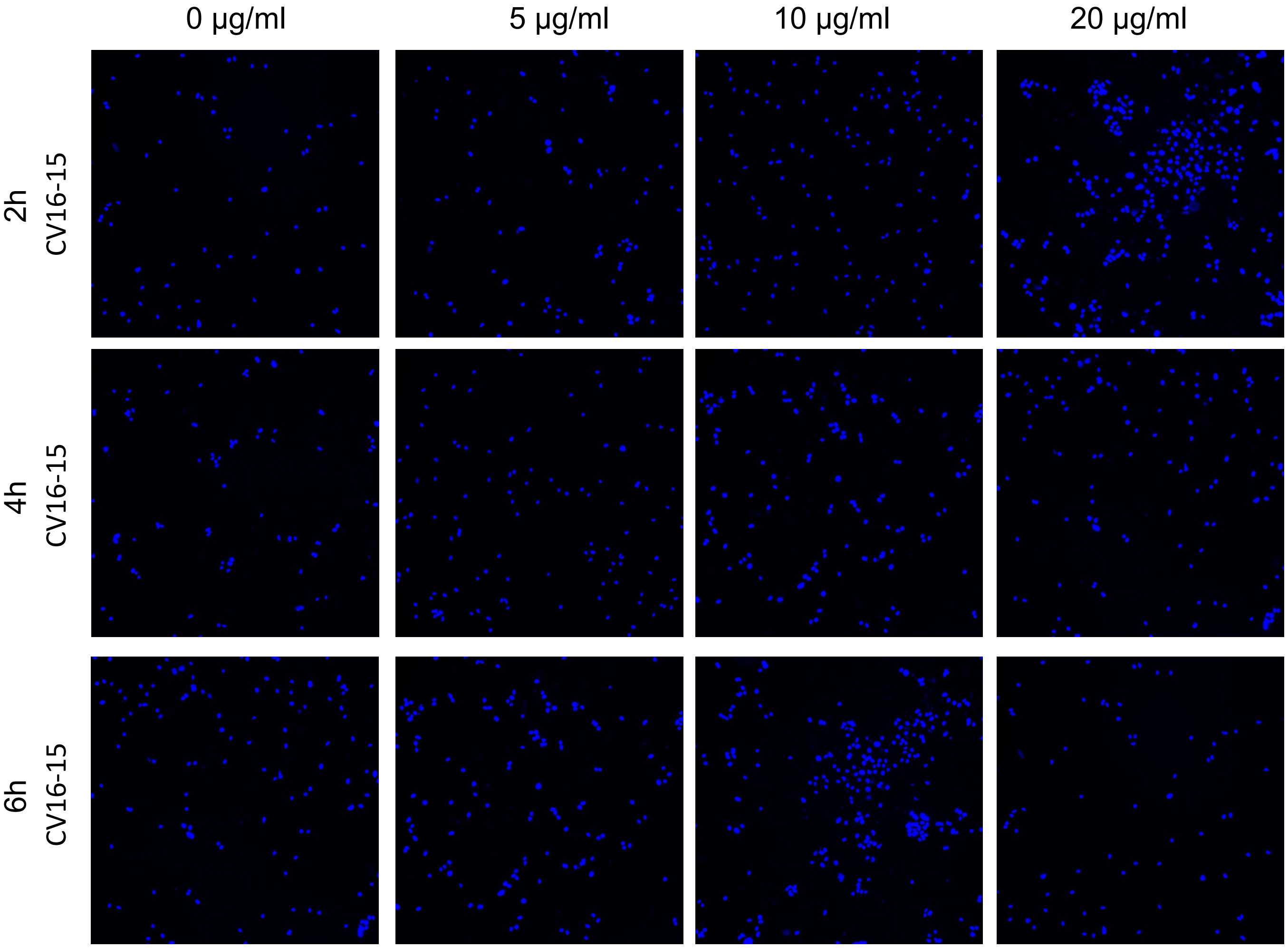

Supplementary Figure S5: gene expression analysis

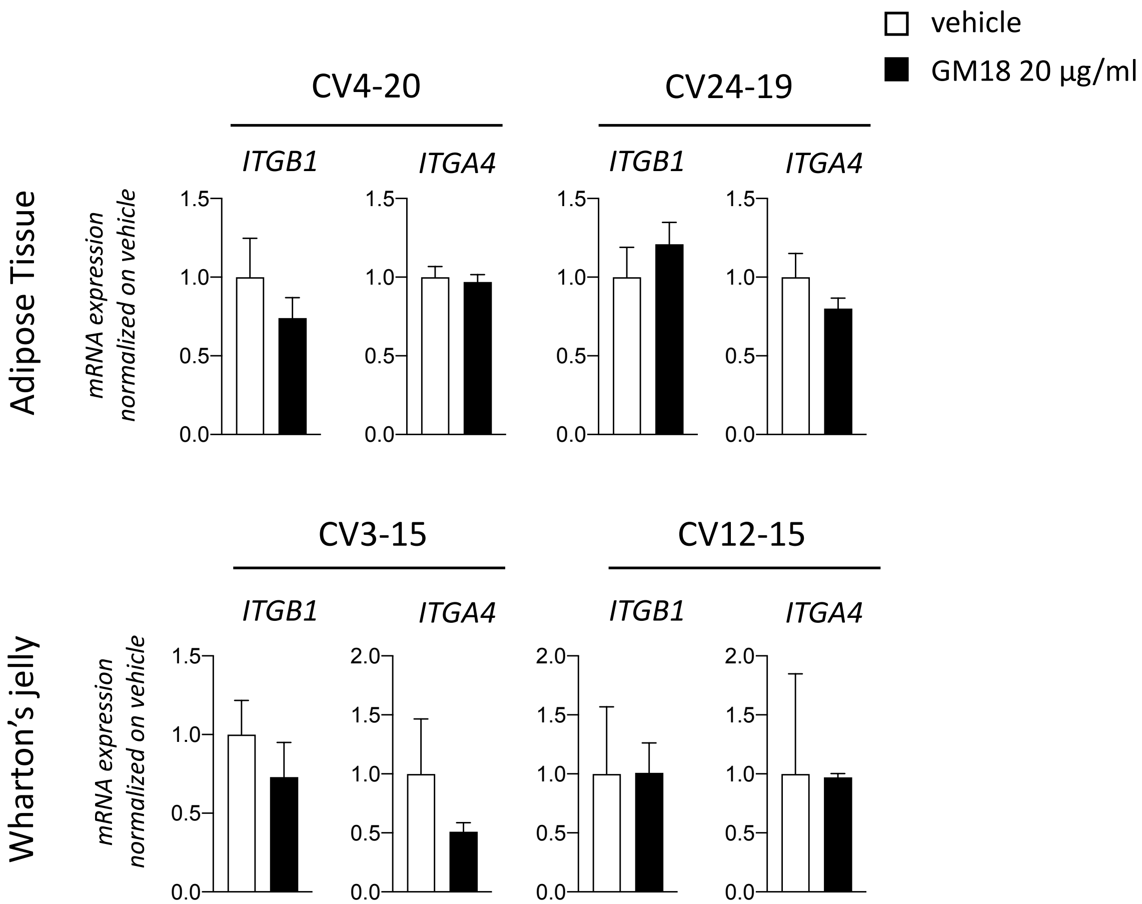

Supplementary Figure S6: HCS analysis of control cultures on glass coverslips

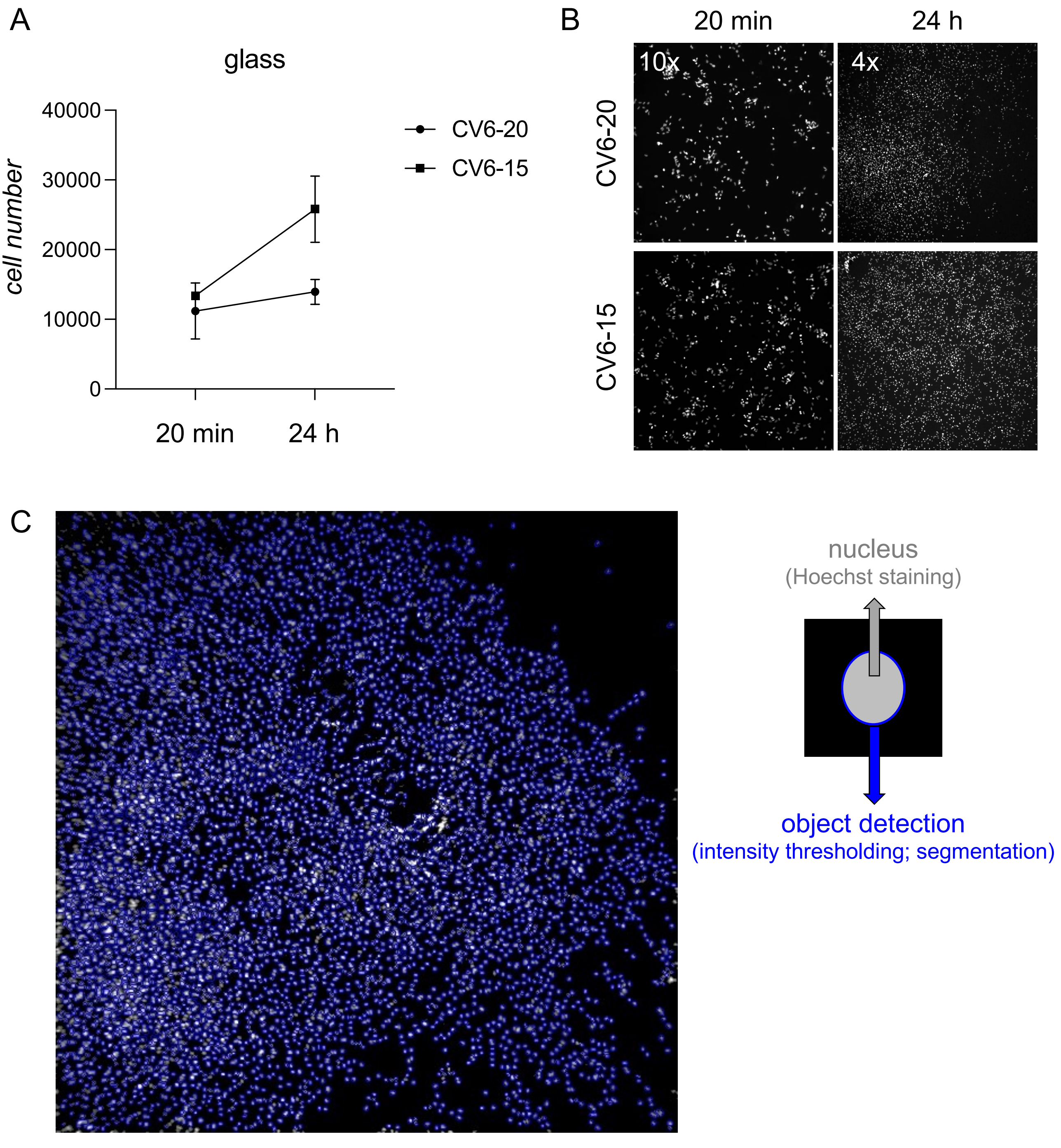

Supplement: Supplementary file 1 [file animals-12-00734-s001.zip › animals-1572856-supplementary.pdf]
